# Implementation of a diagnostic stewardship intervention to improve blood-culture utilization in 2 surgical ICUs: Time for a blood-culture change

**DOI:** 10.1017/ice.2023.249

**Published:** 2024-04

**Authors:** Jessica L. Seidelman, Rebekah Moehring, Erin Gettler, Jay Krishnan, Lynn McGugan, Rachel Jordan, Margaret Murphy, Heather Pena, Christopher R. Polage, Diana Alame, Sarah Lewis, Becky Smith, Deverick Anderson, Nitin Mehdiratta

**Affiliations:** 1 Duke Center for Antimicrobial Stewardship and Infection Prevention Durham, North Carolina; 2 Division of Infectious Diseases, Department of Medicine, Duke University, Durham, North Carolina; 3 Department of Surgery, Duke University Medical Center, Durham, North Carolina; 4 Department of Pathology, Duke University School of Medicine, Durham, North Carolina; 5 Department of Anesthesiology, Division of Anesthesia Critical Care and GVT, Duke University School of Medicine, Durham, North Carolina

## Abstract

**Objective:**

We compared the number of blood-culture events before and after the introduction of a blood-culture algorithm and provider feedback. Secondary objectives were the comparison of blood-culture positivity and negative safety signals before and after the intervention.

**Design:**

Prospective cohort design.

**Setting:**

Two surgical intensive care units (ICUs): general and trauma surgery and cardiothoracic surgery

**Patients:**

Patients aged ≥18 years and admitted to the ICU at the time of the blood-culture event.

**Methods:**

We used an interrupted time series to compare rates of blood-culture events (ie, blood-culture events per 1,000 patient days) before and after the algorithm implementation with weekly provider feedback.

**Results:**

The blood-culture event rate decreased from 100 to 55 blood-culture events per 1,000 patient days in the general surgery and trauma ICU (72% reduction; incidence rate ratio [IRR], 0.38; 95% confidence interval [CI], 0.32–0.46; *P* < .01) and from 102 to 77 blood-culture events per 1,000 patient days in the cardiothoracic surgery ICU (55% reduction; IRR, 0.45; 95% CI, 0.39–0.52; *P* < .01). We did not observe any differences in average monthly antibiotic days of therapy, mortality, or readmissions between the pre- and postintervention periods.

**Conclusions:**

We implemented a blood-culture algorithm with data feedback in 2 surgical ICUs, and we observed significant decreases in the rates of blood-culture events without an increase in negative safety signals, including ICU length of stay, mortality, antibiotic use, or readmissions.

Diagnostic stewardship can be described as interventions prioritizing the right test, for the right patient, to prompt the right action. To improve patient outcomes, diagnostic stewardship of microbiology tests seeks to improve appropriateness of antimicrobial use, to reduce antimicrobial resistance, to deprioritize or limit tests when appropriate, and to reduce costs associated with unnecessary tests.^1,2^ Blood cultures are often ordered for clinical scenarios with low risk of bacteremia.^3–7^ Unnecessary blood cultures that yield false-positive results can increase antibiotic days of therapy, can require additional imaging studies, can increase lengths of stay, and can cause unnecessary removal of indwelling devices.^8^ The 2023 diagnostic stewardship guidelines recommend developing strategies for optimal practices of blood cultures given the implications for patients and hospitals.^9^

The DISTRIBUTE study by Fabre et al^10^ evaluated the implementation of a blood-culture diagnostic stewardship program in a medical intensive care unit (ICU) and 5 medicine units. These researchers reported a decrease in blood-culture rates without significant changes in Centers for Medicare and Medicaid Services (CMS) SEP-1 blood-culture component compliance, all-cause in-hospital and 30-day mortality, and 30-day readmission. Another study by Woods-Hill et al^11^ found that implementation of a blood-culture algorithm in 14 pediatric ICUs across the United States reduced both blood-culture use and antibiotic use.

To the best of our knowledge, no one has published data on blood-culture diagnostic stewardship interventions in adult surgical ICUs. We implemented a blood-culture algorithm as part of a quality improvement initiative to improve blood-culture utilization in 2 surgical ICUs. The goal of the study was to measure effectiveness of the intervention on blood-culture utilization rates as well as patient outcomes, including ICU length of stay (LOS), mortality, and unplanned readmissions.

## Methods

### Setting and study population

Duke University Hospital is a 1,048-bed academic hospital in Durham, North Carolina. The intervention took place in 2 surgical intensive care units: a 24-bed general surgery and trauma ICU and a 32-bed cardiothoracic surgery ICU. The participating units were staffed by resident physicians and advanced practice providers. Patients in the general surgery ICU were generally from general surgery, trauma, transplant, and vascular surgical services. Patients in the cardiothoracic surgery ICU were patients who had had cardiac bypass, valve surgery, lung or heart transplantation, or mechanical circulatory support device insertion.

We implemented a quality improvement intervention in February 2022 in the general surgery and trauma ICU and in March 2022 in the cardiothoracic ICU. We compared blood-culture rates from those of February 2020–January 2022 to those of February 2022–February 2023 in the general surgery/trauma ICU and from those of February 2020–February 2022 to those of March 2022–February 2023 in the cardiothoracic ICU. We compared outcomes for the other adverse events (eg, readmission, death, and days of antibiotic therapy) for both units during the 13-month intervention period (February 2022–February 2023) to outcomes from the preintervention period (February 2020–January 2022). Blood cultures were included if a patient was at least 18 years of age and had been admitted to one of the surgical ICUs when the blood culture was ordered. We excluded blood cultures ordered prior to ICU admission. Although included in the analysis, we advised against applying the blood-culture algorithm to patients with an absolute neutrophil count <500 cells/µL or recipients of a heart or lung transplant. This study was approved by the Duke Institutional Review Board.

### Definition

We defined a blood-culture event as collection of a blood-culture set or blood-culture sets ordered by a clinician for a specific clinical indication. For example, 2 sets of blood cultures drawn for a fever on hospital day 3 were counted as 1 blood-culture event. A blood culture was considered inappropriate if the clinician did not follow the blood-culture algorithm.

Our primary outcome was the blood-culture event rate (blood-culture events per 1,000 patient days) ratio before and after the intervention in each surgical ICU. Our secondary outcomes were average length of stay in the ICU (days), mortality percentage, and unplanned readmission percentage before compared to after the introduction of the algorithm.

### Intervention

**Figure 1.**
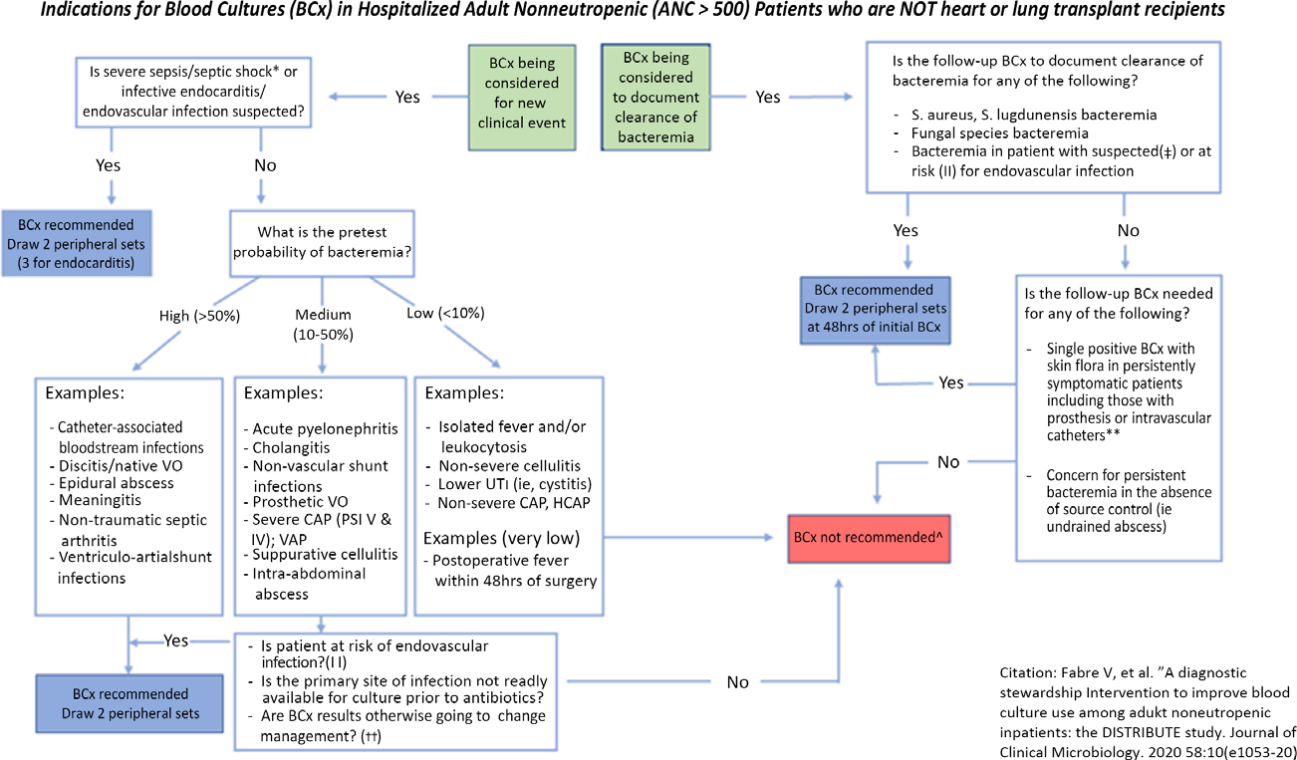
Algorithm to help guideclinical providers when to obtain blood culture for new clinical events or documentation of prior bacteremia clearance.

We assembled a multidisciplinary study team of intensivists, infectious diseases physicians, and nurses to create our institutional blood-culture algorithm based on previous work by Fabre et al.^10^ We implemented this algorithm to guide providers on when to obtain a blood culture, specifically for a new clinical event or for documentation of clearance of prior bacteremia (Fig. 1). Education occurred at the beginning of blood-culture algorithm implementation (February 2022 for the general surgery and trauma ICU, March 2022 for the cardiothoracic ICU) for the consistent providers within each ICU (attending physicians and advanced practice providers). These providers received electronic communication as well as algorithms posted throughout the ICU. They also received frequent reminders through email and in-person feedback during the implementation year. Monthly education occurred for those rotating through these ICUs (fellows and residents), and this group received electronic communication a week prior to their rotation to keep the algorithm pertinent. We implemented the algorithm in the surgical ICU on February 1, 2022, and in the cardiothoracic surgical ICU on March 1, 2022. Electronic reports containing total number of blood-culture events, clinical indications for those blood-culture events, and the proportion of appropriate and inappropriate blood-culture events were provided weekly to the unit medical directors. The medical directors were asked to report any adverse events potentially related to the use of the blood-culture algorithm to the principal investigators.

### Data collection

We reviewed all blood cultures collected in the surgical ICUs using an existing surveillance database. The database contained patient age, admission date, blood-culture date, ordering provider, maximum white blood cell count on the day of blood-culture collection and maximum temperature recorded on the day of blood-culture collection. These data populated a shared spreadsheet accessed by case reviewers. Study team members consisted of 3 infectious disease physicians, 1 anesthesiologist, and 3 surgical nurse practitioners. The team reviewed the electronic medical record to determine the clinical indication for the blood culture and adjudicate if the indication followed the algorithm. If the adjudication could not be made, the record was subjected to a secondary review by a different adjudicator until consensus was reached.

### Statistical analysis

We performed an interrupted time-series analyses for each unit using Poisson regression model to compare rates of blood-culture events (ie, blood-culture events per 1,000 patient days) before and after the algorithm implementation. We defined β1 as the slope in the preintervention period, β2 as the slope at the time of the intervention, and β3 as the slope following the intervention. When comparing blood-culture event rates, the preintervention period for the general surgery and trauma ICU included data from February 1, 2020, through January 31, 2022, and for the cardiothoracic surgery ICU from February 1, 2020, through February 28, 2022. Similarly, the intervention group for the general surgery and trauma ICU included data from February 1, 2022, through February 28, 2023, and the intervention group for the cardiothoracic surgery ICU included data from March 1, 2022, through February 28, 2023.

When comparing outcome variables of readmission, mortality, and antibiotic days of therapy for both units, we used a preintervention period of February 1, 2020, through January 31, 2022, compared to an intervention period of February 1, 2022, to February 28, 2023. Categorical variables were compared using χ^2^ tests and continuous variables were compared using the Student *t* test. A 2-sided *P* value <.05 was considered statistically significant. All statistical analyses were performed using SAS version 9.4 software (SAS Institute, Cary, NC).

## Results

### Blood-culture event rates

For the general surgery and trauma ICU, we included 1,542 blood-culture events in 823 unique patients in the preintervention group from February 1, 2020, through January 31, 2022, and 473 blood-culture events in 285 unique patients in the intervention group from February 1, 2022, to February 28, 2023. For the entire general surgery and trauma ICU population (ie, patients with and without blood-culture events), there were 15,365 patient days and 4,323 admissions in the preintervention period and 8,530 patient days and 656 admissions in the intervention period. For the cardiothoracic surgery ICU, we included 2,148 blood-culture events in 810 unique patients in the preadmission period from February 1, 2020, to February 8, 2022, and we included 885 blood-culture events in 347 unique patients in the intervention group from March 1, 2022, to February 28, 2023. For the entire cardiothoracic ICU population, there were 20,931 patient days and 4,482 admissions in the preintervention period and 11,554 patient days and 1,965 admissions in the intervention period. For the preintervention period, the monthly average blood-culture rates were 193 blood-culture events per 1,000 patient days in the general and trauma ICU and 191 blood-culture events per 1,000 patient days in the cardiothoracic ICU. In the postintervention period, the monthly average blood-culture rate dropped to 56 blood-culture events per 1,000 patient days and to 77 blood-culture events per 1,000 patient days, respectively.

**Figure 2.**
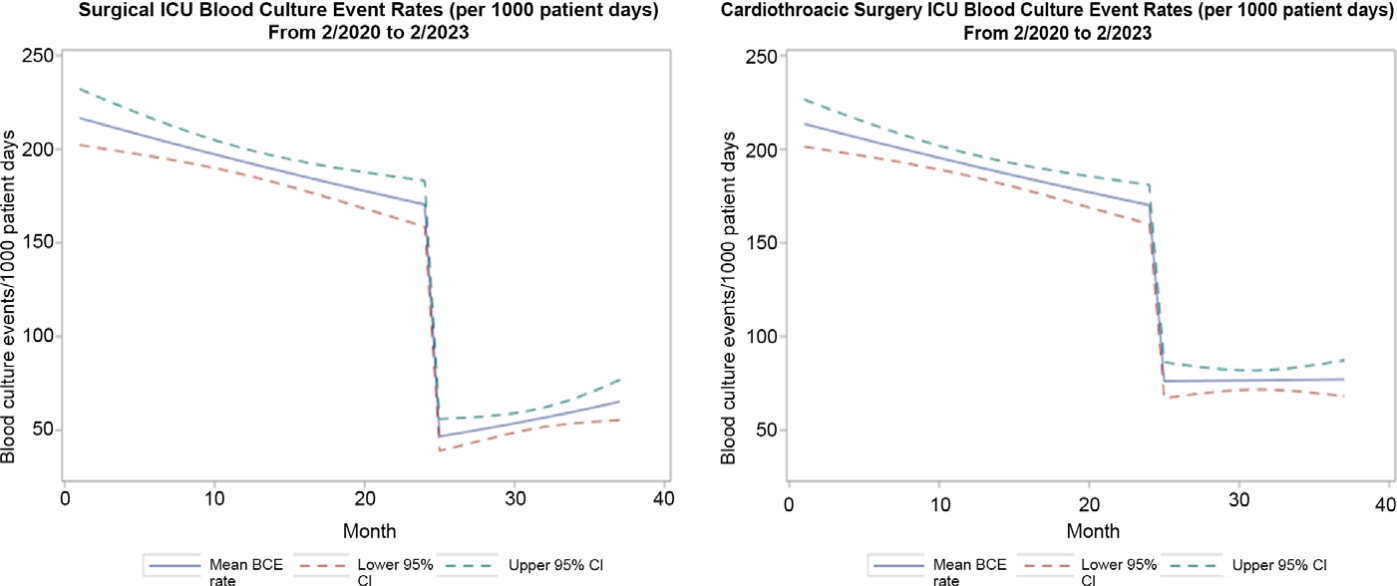
Monthly blood culture event rate (per 1000 inpatient days) for general/trauma ICU before (2/2020-1/2022) and after (2/2022-2/2023) the blood culture algorithm introduction.

In the general and trauma surgical ICU, there were longitudinal reductions in blood-culture events, with β1= −0.010 (95% confidence interval [CI], −0.016 to 0.005; *P* < .01) prior to the intervention (Fig. 2). At the time of the intervention, there was an acute drop, with β2 = −2.24 (95% CI, −3.02 to −1.46; *P* < .01), followed by a slowly increasing slope, with β3 = 0.039 (95% CI, 0.013–0.063; *P* < .01). Despite the slow increase during the months following the intervention, the rate of blood-culture events continued to be significantly lower than during the period before the algorithm was implemented (incidence rate ratio [IRR], 0.38; 95% CI, 0.32–0.46; *P* < .01). Aside from the blood-culture event rates, we did not find statistically significant differences between the preintervention and intervention groups regarding patient age, days from admission to blood-culture event, the maximum white blood cell count (WBC) on the day of the blood-culture event, nor the maximum temperature on the day of the blood-culture event (Table 1).

**Table 1.** Characteristics of Patients with Blood Cultures and Characteristics of Blood-Culture Events in the General and Trauma Surgery ICU Before and After Implementation of a Blood-Culture Algorithm

Variable	Preintervention Period	Intervention Period	*P* Value
No. of blood-culture events	1,543	473	
No. of patients	822	285	
Patient age, median y (IQR)	59 (45–70)	59 (47–67)	.28^a^
Blood-culture events per 1,000 patient days	100	55	<.01^b^
Days from admission to blood-culture event, median (IQR)	8 (3–15)	7 (2–19)	.06^c^
Maximum WBC on day of blood-culture event, mean ×10^9^ cells/L (SD)	15.3 (9.0)	17.1 (10.5)	.49^c^
Max temperature on day of blood-culture event, °F (SD)	100.4 (1.7)	100 (1.6)	.10^c^

Note. ICU, intensive care unit; IQR, interquartile range; WBC, white blood cell count; SD, standard deviation.

a Kruskal-Wallis test.

b χ^2^ test.

c *t* test.

In the cardiothoracic surgery ICU, our investigation yielded similar findings. Prior to the intervention, there were longitudinal reductions in blood-culture events, with β1= −0.010 (95% CI, −0.014 to −0.005; *P* < .01) (Fig. 2). At the time of the intervention, there was an acute drop, with β2 = 1.07 (95% CI, −1.63 to −0.51; *P* < .01), followed by a slowly increasing slope, with β3 = 0.011 (95% CI, −0.007 to 0.029; *P* = .24). Despite the slow increase during the months following the intervention, the rate of blood-culture events continued to be significantly lower than during the period before the algorithm was implemented (IRR, 0.45; 95% CI, 0.39–0.52; *P* < .01). Other than the blood-culture event rate, the patients with blood-culture events were significantly older in the intervention group compared to the preintervention group, but this was not clinically relevant. Similar to the general and trauma surgery ICU, the following factors were not significantly different between the preintervention and intervention groups: days from admission to blood-culture event, the maximum white blood cell count (WBC) on the day of the blood-culture event, nor the maximum temperature on the day of the blood-culture event (Table 2).

**Table 2.** Characteristics of Patients with Blood Cultures and Characteristics of Blood-Culture Events in the Cardiothoracic Surgery ICU Before and After Implementation of a Blood-Culture Algorithm

Variable	Preintervention Period, No. (%)^a^	Intervention Period, No. (%)^a^	*P* Value
Blood-culture events	2,147	885	
No. of patients	811	347	
Patient age, median y (IQR)	59 (48–68)	63 (51–71)	.02^b^
Blood-culture events per 1,000 patient days	103	77	<.01^c^
Days from admission to blood-culture event, median d (IQR)	12 (5–28)	15 (6–29)	.98^d^
Maximum WBC on day of blood-culture event, mean ×10^9^ cells/L (SD)	18.5 (9.9)	19.3 (9.8)	.39^d^
Max temperature on day of blood-culture event, °F (SD)	99.6 (1.3)	99.6 (1.6)	.09^d^

Note. WBC, white blood cell count; IQR, interquartile range; WBC, white blood cell count; SD, standard deviation.

a No. (%) unless otherwise indicated.

b Kruskal-Wallis test.

c χ^2^ test.

d *t* test.

### Blood-culture indications and appropriateness

For the period from February 2022 to February 2023, we reviewed a total of 1,237 blood-culture events (Table 3). The reviewed blood-culture event cohort consisted of 772 men (62%) and 465 women (38%). Among the reviewed blood-culture events, 86 occurred in liver transplant recipients, 20 occurred in multivisceral transplant recipients, 19 occurred in kidney transplant recipients, and 1 occurred in a small bowel transplant recipient. The most common reasons for blood-culture events were sepsis or septic shock (466, 37.7%), isolated fever and/or leukocytosis (288, 18.4%), and documenting clearance of bacteremia (183, 14.8%) (Table 3). Overall, 876 (70.8%) blood-culture events were deemed to follow the blood-culture algorithm. The most common clinical scenario in which a blood-culture event did not follow the blood-culture algorithm was for isolated fever and/or leukocytosis (228, 54.3%) and documenting clearance of bacteremia (28, 7.8%).

**Table 3.** Distribution of Reviewed Blood-Culture Events by Clinical Indication and Further Stratified by whether the Clinical Indication Followed the Blood-Culture Algorithm (Appropriate) or Not (Inappropriate)

Indication	Total, No. (% of All Indications)	Appropriate Blood Culture, No. (% of Row)	Inappropriate Blood Culture, No. (% of Row)
Severe sepsis or septic shock	466 (37.7)	466 (100)	0 (0)
Isolated fever and/or leukocytosis	228 (18.4)	0 (0)	228 (100)
Documenting clearance of bacteremia	183 (14.8)	155 (84.7)	28 (15.3)
Other^a^	179 (14.5)	104 (58.1)	75 (41.9)
Suspected infective endocarditis or endovascular infection	68 (5.5)	68 (100)	0 (0)
Ventilator-associated pneumonia	51 (4.1)	49 (96.1)	2 (3.9)
Post-op fever within 48 h of surgery	18 (1.5)	0 (0)	18 (100)
Severe community-acquired pneumonia	9 (0.7)	7 (77.8)	2 (22.2)
Catheter-associated bloodstream infection	8 (0.7)	8 (100)	0 (0)
Nonsevere community-acquired pneumonia or hospital-acquired pneumonia	7(0.6)	3 (42.9)	4 (57.1)
Severe cellulitis or cellulitis in patient with comorbidities	6 (0.5)	4 (66.7)	2 (33.3)
Cholangitis	4 (0.3)	4 (100)	0 (0)
Acute pyelonephritis	3 (0.2)	2 (66.7)	1 (33.3)
Native septic arthritis	3 (0.2)	2 (66.7)	1 (33.3)
Meningitis	2 (0.1)	2 (100)	0 (0)
Discitis/native vertebral osteomyelitis	1 (0.1)	1 (100)	0 (0)
Lower urinary tract infection (cystitis or prostatitis)	1 (0.1)	1 (100)	0 (0)
Total	1237	876 (70.8)	361 (29.2)

a Examples of “other” indication include bacteremia in a donor at the time of organ donation, intra-abdominal abscess, mediastinitis.

### Outcome measures

Outcome measures for all patients admitted to 1 of the 2 surgical ICUs during the preintervention and intervention periods are described in Table 4. This cohort was not limited to patients who had a blood-culture event during the hospitalization. We found no significant differences in the ICU length of stay (days), in-hospital mortality, 30-day all-cause mortality rate, and 30-day all cause readmission rate between the preintervention and intervention periods. The average monthly antibiotic days of therapy (2,162 vs 2,032; *P =* .03) and 90-day all-cause mortality rate (14.1% vs 12.7%; *P* = .04) were significantly lower following the intervention. Lastly, the central-line–associated bloodstream infection (CLABSI) rates for both surgical ICUs did not change significantly with the blood-culture algorithm intervention (relative risk [RR], 0.83; *P* = .50) (Table 4).

**Table 4.** Outcomes Measures Among the Patients who were Admitted to One of the Surgical ICUs in the Preintervention and Postintervention Periods^a^

Outcome Measure	Preintervention Period (N=7,316), No. (%)^b^	Intervention Period (N=3620), No. (%)^b^	*P* Value
ICU LOS days (SD)	5.0 (9.6)	5.4 (10.2)	0.22^c^
Average monthly antibiotic days of therapy (SD)	2,162 (148.5)	2032 (173.7)	0.03^c^
In-hospital mortality	676 (9.2)	319 (8.8)	0.46^c^
30-d all-cause mortality	872 (11.9)	387 (10.7)	0.06^d^
90-d all-cause mortality	1034 (14.1)	460 (12.7)	0.04^d^
30-d unplanned readmission	829 (11.3)	398 (11.0)	0.85^d^
CLABSI events	53	17	
CLABSIs per 1,000 central-line days	1.22	1.04	0.50^d^

Note. ICU, intensive care unit; LOS, length of stay; SD, standard deviation; CLABSI, central-line–associated bloodstream infection.

a Includes patients who had a blood-culture event and those who did not.

b No. (%) unless otherwise indicated.

c *t* test.

d χ^2^ test.

## Discussion

By introducing a blood-culture algorithm as a part of a diagnostic stewardship intervention in a general and trauma surgical ICU and cardiothoracic surgery ICU, we reduced the blood-culture event rate by 45% and 25%, respectively, without an increase in any negative counterbalancing measures that we were able to evaluate. The difference in blood-culture event reduction between the 2 surgical ICUs is likely explained by the presence of heart- and lung-transplant recipients in the cardiothoracic ICU, to which the blood-culture algorithm was not applied.

In the surgical population, there is a high incidence of fever and leukocytosis following trauma or surgery due to noninfectious causes. The blood-culture algorithm specifies that for isolated leukocytosis and/or fever, blood cultures should not be drawn due to a low risk of bacteremia. Patients in the surgical ICUs likely benefitted the most from discouraging blood cultures in those with isolated fever and/or leukocytosis. As such, this study likely represents a unique patient cohort compared to the medical ICU patients and medical floor patients described in prior work.

Our study had a few differences from the prior DISTRIBUTE study by Fabre et al.^10^ Our intervention took place in 2 surgical ICUs compared to the prior work in which the blood-culture algorithm was implemented in 1 medical ICU and 5 medicine units. We found a higher blood-culture event rate reduction in our surgical ICUs (45% in general and trauma surgical ICU and 25% in the cardiothoracic surgery ICU) compared to the 18% reduction found in the medical ICU in the former study. Although we did have a similar proportion of appropriate initial blood-culture events compared to Fabre et al in the ICUs (71.5% vs 75%), we had a higher proportion of appropriate follow-up blood-culture events for documentation of bloodstream clearance in the ICUs (84.7% vs 54%). Lastly, we reviewed every blood culture collected in both surgical ICUs during the intervention period as opposed to a random sample of blood cultures, as done by Fabre et al.

We encountered some challenges in implementing the blood-culture algorithm. Ordering blood cultures is part of an ingrained practice in medicine when a patient has a fever or leukocytosis. We encountered some hesitancy with acceptance of the algorithm among transplant infectious disease providers and surgical consulting services. One issue was that the education intervention was targeted to providers in the surgical ICUs, and often these patients were comanaged by multiple surgical services and consulting teams. Additionally, there was a dearth of data on how to optimize blood-culture diagnostic stewardship among transplant-recipient patients and patients on immunomodulating therapies. Lastly, the intervention of reviewing all blood-culture events each week was labor intensive and not sustainable for our team after the study period. In fact, we encountered many of the same challenges in implementing blood-culture stewardship to those described in antibiotic stewardship rounding regarding efficiency, acceptance, and education.

Although initial implementation of the algorithm was met with a significant decrease in blood-culture event rate in both ICUs, we noted a gradual increase in blood-culture events during the intervention period. This situation is likely multifactorial. First, the underlying practice of drawing blood cultures for clinical scenarios that include low probability of bacteremia is a longstanding and deep-rooted convention. Second, we had to education and orient new trainees to the algorithm and change in practice every month. Third, the process of reviewing every blood culture and adjudicating their appropriateness was time intensive. We had difficulty maintaining enthusiasm for this practice after several months.

Our study had several limitations. We did not assess whether a positive blood culture was a contaminant or a true positive. We assumed that the contamination rate was similar in the preintervention and postintervention periods given that no other significant interventions among blood-culture collection occurred during the study period. The study was only performed in a single, tertiary-care, academic medical center, which limits the generalizability of these findings to other clinical contexts. Next, the clinical indication was ascertained by retrospective chart review. As such, misclassification bias may have occurred if a blood culture was ordered for an appropriate reason but the documentation was not provided to support the reason behind ordering the cultures. Lastly, the outcome measures applied to all patients admitted to the 2 units during the study period rather than just patients whose diagnostic workup would be eligible for consideration in the blood-culture algorithm. This factor may have biased toward the null, though we note the small number of transplant recipients included in blood-culture event reviews (n = 126, 10.2% of reviewed).

Next, we would like to study the application of the blood-culture algorithm in patients who are immunocompromised, including transplant recipients and those receiving immunomodulating therapy. We would also like to evaluate methods to reinforce diagnostic stewardship that are not as labor intensive. Specifically, we would like to investigate the use of a best-practices alert in the electronic medical report to prompt reassessments of ordering blood cultures for clinical scenarios with low risk of bacteremia. In addition, we would also like to look at the “drift” in blood-culture ordering after the intervention of reviewing every blood culture for appropriateness has ended. This would make the outcomes of a blood-culture algorithm more generalizable.

In conclusion, we present a prospective cohort study of a diagnostic stewardship intervention using a blood-culture algorithm in 2 surgical ICUs in which we reduced blood-culture utilization rates and did not observe any patient harm.
